# Missed Opportunities to Promptly Diagnose and Treat Polyradiculoneuropathy and Acute Motor Neuropathy: A Case Study

**DOI:** 10.7759/cureus.54068

**Published:** 2024-02-12

**Authors:** Hyppolite Tchidjou K, Cécile Grenenko

**Affiliations:** 1 Pediatrics, Pediatric Emergency Services, University Hospital of Amiens Picardie, Amiens, FRA; 2 Pediatric Neurology Services, University Hospital of Amiens Picardie, Amiens, FRA

**Keywords:** treatment, electromyography, polyradiculoneuropathy, children, neuropathy

## Abstract

Despite progress, the diagnosis and relative management of inflammatory demyelinating polyradiculoneuritis with motor and sensory involvement remain challenging in non-specialized medical centers.

We describe the case of a seven-year-old girl admitted to our hospital, with asthenia, headaches, mild diffuse pain, gait disturbances, and decreased strength of lower limbs. On admission to our hospital center, in addition to the blood tests and the cerebral and medullary magnetic resonance imaging, we performed an analysis of her cerebrospinal fluid. This test confirmed an albuminocytological dissociation and electromyography, which suggested the diagnosis of acute inflammatory demyelinating polyneuropathy with motor and sensory involvement. The child was successfully treated with polyvalent immunoglobulin and neuromotor physiotherapy.

This report highlights the importance for the clinician not to focus only on their protocols training but also on the ability to relate correctly the results of the required tests, to the patient's symptoms.

## Introduction

Polyradiculoneuropathy is a rare neurological disorder characterized by an autoimmune process that damages the myelin encasing of the peripheral nerve. In many cases, the exact cause stays unknown [1,2]. 

Clinically, is characterized by paresthesia, a gradual weakening, loss of feeling and reflexes in the arms and legs, but also loss of balance and ability to walk and often evolves to ascending paralysis, depending on the common type or variant. On a laboratory level, we can observe an albuminocytological dissociation in the cerebrospinal fluid (CSF) analysis, even if a normal CSF protein level does not rule out such diagnosis, because it could also depend on the time of onset disease and lumbar puncture [2,3].

Patients with symptoms and signs suggesting neuropathy generally should undergo nerve conduction studies as well as electromyography (NCS/EMG), spinal tap, and blood tests, which is the primary diagnostic tool in the evaluation of patients with large-fiber polyneuropathy. NCS/EMG helps clinicians categorize polyneuropathy as primary axonal versus primary demyelinating [3,4]. In the last years, magnetic resonance imaging, neuromuscular ultrasound, and serological testing for antibodies have also shown promising results in the assessment of peripheral neuropathy [5].

We report a case of a seven-year-old girl child with a delayed diagnosis of peripheral neuropathy, who was successfully treated during hospitalizations with four doses of intravenous immunoglobulin.

## Case presentation

A seven-year-old girl was admitted in June 2022 to the pediatric emergency services (PES) of the University Hospital of Amiens due to asthenia, headaches, and lower limb weakness three weeks ago, followed by gait disorders accompanied by diffuse pain without paresthesia.

The patient had a medical history of obesity, transient benign neutropenia, asthma, and sacrococcygeal dimple operated. Her family history was unremarkable. She was up to date with the immunization schedule. A few weeks before admission, she had an episode of strep throat treated, and a few days before the symptoms appeared she reported one to two days of acute gastroenteritis.

At admission, neurological examination revealed waddling gait, positive Gowers, difficulty tiptoeing, numbness, and unsteadiness of lower limbs, and the Babinski sign was present even in the absence of other pyramidal system disorders. The joints had a full range of movement with no swelling or tenderness. She did not show any signs of trauma, redness or rashes, edema, or warmth to the limbs. The remainder of the physical examination revealed no abnormalities. No fever or cognitive function deterioration was mentioned, and her neurological development was normal.

The patient had first been hospitalized at a regional hospital in Somme - France. During this first hospitalization, initial basic laboratory tests were normal (Table 1). The urine strip and cytology exam of urine were negative. A nasopharyngeal swab for severe acute respiratory syndrome coronavirus 2 (SARS‑CoV‑2) by real-time reverse-transcriptase polymerase-chain-reaction (rRT-PCR) assay was negative. Aerobic and anaerobic blood cultures tested negative. The serologic tests were negative for the herpes virus, Epstein-Barr virus, Cytomegalovirus, Yersinia, and Lyme disease.

**Table 1:** Clinical features, investigations, and therapies Basic Laboratory: normal value (NV), cerebrospinal fluid (CSF), basic laboratory exam (complete blood count “CBC”, C-reactive protein “CRP”, erythrocyte sedimentation rate “ESR”, and leukocyte count, electrophoresis of serum proteins, renal and liver markers), urine strip, and cytology exam of urine Virology and Microbial Serology Tests: SARS-CoV-2, aerobic and anaerobic blood cultures, herpes virus, Epstein-Barr virus, Cytomegalovirus, Hepatitis B and C, varicella-zoster virus, parvovirus B-19, toxoplasmosis, enteroviruses, respiratory viruses, Lyme disease, and Mycoplasma pneumoniae Lumbar Puncture: CSF white cell count and cultures for both bacterial and virus: Enterovirus ribonucleic acid, human herpes virus 6 (HHV-6), herpes simplex virus (HSV), and adenovirus Immunohistochemistry and Hormonal Tests: Ammonia and lactate, rheumatoid factor and complement study, antinuclear antibodies and anti-neutrophil cytoplasmic antibodies (ANCA), anti-cardiolipids, carnitine assay and acylcarnitine profile, urinary organic acid chromatography, anti-ganglioside antibodies, cortisol, adrenocorticotropic hormone (ACTH), insulin-like growth factor 1 (IGF-1), anti-acetylcholine receptor, and anti-MUSK antibodies. Anti-central nervous system antibodies, anti-onconeuronal antibodies, anti-aquaporin 4 antibody, antibody myelin oligodendrocyte glycoprotein, and acetylcholine receptor antibody Imaging: abdominal ultrasonography (US), brain and spine magnetic resonance imaging (MRI) Electromyography (EMG): in the upper and lower limbs

Admission	Clinical features	Investigations	Results	Treatment
Hospitalization at Regional Hospital, May 2022	- Waddling gait, - Positive Gowers, - Difficult to tiptoe, - Numbness, unsteadiness of lower limbs, - Positive Babinski on the left lower limb	Basic Laboratory and Urine Tests	Normal	Clinical monitoring
		Virology and Microbial Serology Tests	Negative	
		Lumbar Puncture	Protein 1.37 g/l; Glucose 4.2 mmol/L; Cell count 5/mm3	
		Imaging (US, MRI)	Normal	
Hospitalization at University Hospital, June 2022	- Numbness, unsteadiness of the lower limbs, - Positive Gowers, - Headaches	Basic Laboratory Tests	Normal	Intravenous immunoglobulin for two days: 30g daily dose Neuromotor physiotherapy
		Serologic Tests	Negative	
		Antistreptolysin O (ASO)	385 UI/ml (nv < 214 UI/ml)	
		Throat Swab	Negative	
		Lumbar Puncture	Protein 1.34 g/l	
		Immunohistochemistry and Hormonal Tests	Normal	
		Electromyography (EMG)	Motor conduction velocities of the right common fibular nerve at 33.6 m/s and the right tibial nerve at 39.4 m/s.	
Hospitalization at University Hospital, July 2022	- Persistence of numbness and unsteadiness of the lower limbs - Difficulties with static and dynamic balance, especially when jumping - Positive Gowers - Headaches	Basic Laboratory	Normal	Intravenous immunoglobulin for two days: 30g daily dose, neuromotor physiotherapy
		Lumbar Puncture	Protein 0.76 g/l	
		Antistreptolysin O (ASO)	341 UI/ml (nv < 214 UI/ml)	
		Outpatients, October 2023	- Improved gross motor skills, - Persistence of osteotendinous hyporeflexia		Electromyography (EMG)	Normalization of latencies and amplitudes for the motor nerves tested, compared to the previous exam	Neuromotor physiotherapy
Outpatients, January 2023	- Normal clinical exam	Electromyography (EMG)	Normal	Clinical monitoring

Lumbar puncture revealed cerebrospinal fluid (CSF) protein 1.37 g/l (reference range 0.15-0.45 g/l), with normal CSF white cell count. CSF cultures were sterile (both bacterial and virus: Enterovirus ribonucleic acid, human herpes virus 6 (HHV-6), herpes simplex virus (HSV), and adenovirus). Abdominal ultrasonography (US), brain magnetic resonance imaging (MRI) with and without contrast, and whole spine MRI were normal (Table 1).

Two days after hospitalization (May 25, 2022), due to partial clinical improvement, the patient was discharged without treatment with lameness diagnosis and clinical monitoring, without EEG or EMG because they were not available in that hospital.

One week after discharge, during the follow-up visit to her family doctor, the patient was referred to the PES of the University Hospital of Amiens due to the persistence of lower limb weakness and gait disorders. At admission, clinical examination revealed a waddling gait with hyperlordosis, positive Gowers, difficulty tiptoeing, numbness, and unsteadiness of lower limbs, Babinski sign present on the left, no ataxia, no dizziness, nausea and vomiting, and no headache. Examination of cranial nerves (CNs), sphincter function, deep sensations, and sensory innervation were normal, and after a neuropediatrician consultation, she was immediately hospitalized in the Neuropediatrics Unit.

At admission, basic laboratory tests were normal (Table 1). Biochemical laboratory tests with ammonia and lactate, rheumatoid factor and complement study, antinuclear antibodies and anti-neutrophil cytoplasmic antibodies (ANCA), anti-cardiolipids, carnitine assay and acylcarnitine profile, urinary organic acid chromatography, anti-ganglioside antibodies, cortisol, adrenocorticotropic hormone (ACTH), insulin-like growth factor 1 (IGF-1), anti-acetylcholine receptor, and anti-MUSK antibodies, as well as hematological tests, were normal.

The serologic tests revealed no infection for herpes virus, Epstein-Barr virus, Cytomegalovirus, Hepatitis B and C, varicella-zoster virus, parvovirus B-19, toxoplasmosis, enteroviruses, respiratory viruses, Lyme disease, and Mycoplasma pneumoniae.

Antistreptolysin O (ASO) titer was positive at 341 UI/ml (normal value < 214 UI/ml), with a negative throat swab. Blood, urine, stool, and cerebrospinal fluid culture were negative.

Analysis of cerebrospinal fluid confirmed an albuminocytological dissociation (ACD) with protein of 1.34 g/L, leucocytes 5/mm^3^, red blood cells 8/mm^3^, and a normal glucose level.

**Figure 1:**
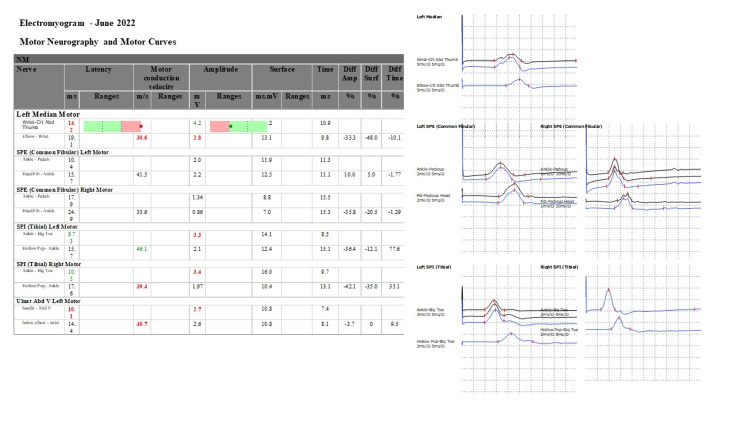
Electromyogram - June 2022

One month after the onset of symptoms, the child underwent nerve conduction studies (NCSs) and Electromyography (EMG) of the upper and lower limbs, which showed reduced motor conduction velocities of the right common fibular nerve at 33.6 m/s and the right tibial nerve at 39.4 m/s (normal value: >40 m/s) with lengthening of latencies in amplitudes and polyphasic appearance raising suspicion of axonal polyradiculoneuritis versus a demyelinating disease (Figure 1). Given the clinical assessment and the patient's symptoms, the neurologist's team made the diagnosis of polyradiculoneuropathy and acute motor neuropathy.

Facial EMG with blink reflex revealed a clear increase in latencies of the fifth and seventh nerves bilaterally and symmetrically, with increased latencies at blink reflex, demyelination, and the axonal part of the face.

Anti-central nervous system antibodies, anti-onconeuronal antibodies, anti-aquaporin 4 antibodies, antibody myelin oligodendrocyte glycoprotein, and acetylcholine receptor antibodies were all normal (Table 1).

One month after the onset of symptoms, due to a diagnostic delay in the local hospital, the patient was treated with intravenous immunoglobulin (1 g/kg weight, daily dose for two days) and neuromotor physiotherapy, with a favorable outcome. Based on significant improvement in numbness and unsteadiness, and with Gowers sign negativization, she was discharged with a diagnosis of polyradiculoneuropathy, acute motor neuropathy, and neuromotor physiotherapy follow-up. 

One month later, given the persistence of numbness and unsteadiness of the lower limbs, she was hospitalized once again and received two other doses in two days at the same dosage (30 g daily dose) of intravenous immunoglobulin and neuromotor physiotherapy support. Three days later, after seeing a full recovery of symptoms, she was discharged without treatment and the team of neurologists confirmed the diagnosis of polyradiculoneuropathy and acute motor neuropathy with an EMG control programmed for follow-up (Table 1).

**Figure 2:**
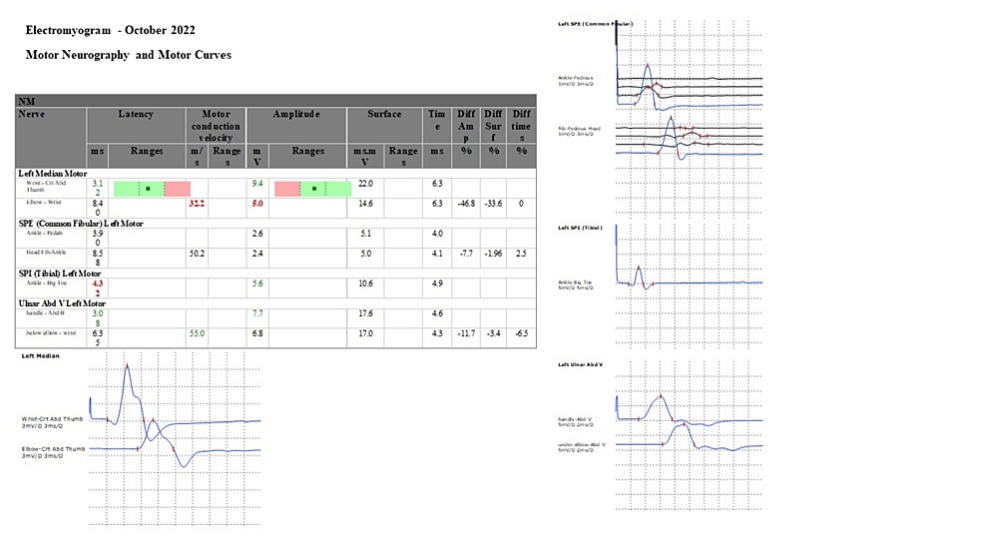
Electromyogram - October 2022

At the outpatients’ follow-up in October 2023, a further clinical improvement was observed and the muscle control test showed good improvement with normalization of latencies and amplitudes for the motor nerves tested, compared to the previous exam (Figure 2).

**Figure 3:**
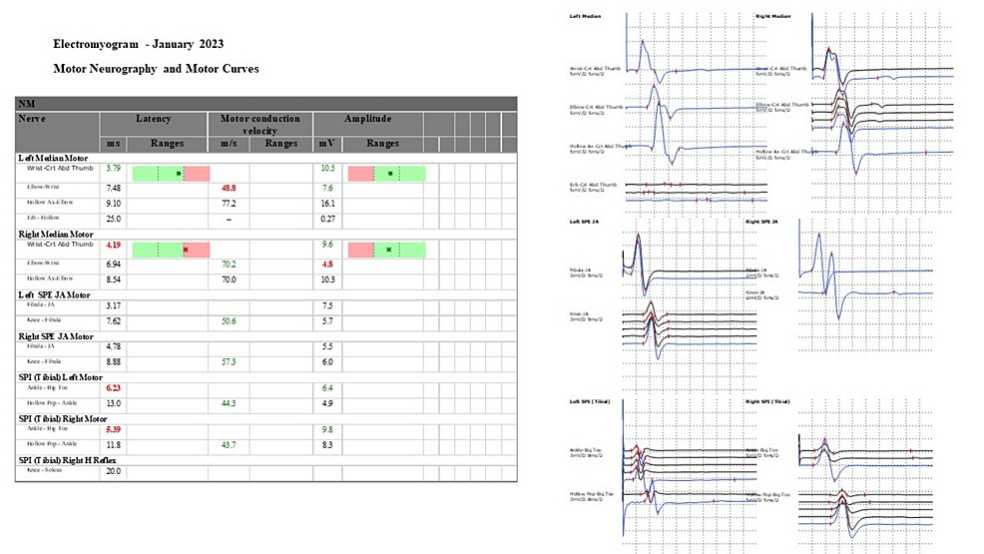
Electromyogram - January 2023

At the outpatient’s follow-up in January 2023, she had fully recovered, the clinical examination was normal, and the muscle test returned to normal/physiological status/without any abnormality (Figure 3).

## Discussion

The management of peripheral neuropathies in children is still a challenge. Recommendations and randomized control trials for children are lacking in recently published guidelines [3].

One of the most common types of peripheral neuropathies is axonal sensory and sensorimotor polyneuropathies, which is an immune-mediated post-infectious neurological disorder with an incidence of 0.5 to 2/100,000 and a male predominance characterized by demyelination of spinal nerve roots and peripheral nerves and an albumin-cytological dissociation in the cerebrospinal fluid (CSF).

Furthermore, patients with this monophasic pathology can, in some cases, have partial recovery, and therefore, they can be hospitalized several times to also rule out other pathologies such as traumatic neuropathies or systemic diseases, especially to check for causes such as diabetes, drug intoxication, or vitamin B12 deficiency. Occasionally, a nerve biopsy and genetic tests may be carried out to exclude genetic abnormalities such as Charcot-Marie-Tooth disease [3,6].

The main etiology is a trigger that induces the generation of autoantibodies targeting peripheral nerve myelin and/or axonal membranes, thus leading to an important shortage in conduction velocities and specific electrophysiological features [2].

The standard treatment for polyradiculoneuropathy and acute motor and axonal neuropathy immunotherapy remains intravenous immunoglobulin (IVIG) administration or plasmapheresis in association with supportive care. It is important to underline that the severe forms could be managed either with IVIG or with rituximab [3].

## Conclusions

Given that these are rare and complex neurological abnormalities, this case report may be helpful for clinicians because it highlights the importance for clinicians not to focus only on the patient's symptoms but also have the ability to correctly relate the results of the required tests to the patient's symptoms to optimize your patient management and request specialist advice where necessary and at the right time. This is especially true when it comes to complex pathologies that need specialized exams such as lumbar puncture and immunohistochemistry tests, electromyography, magnetic resonance imaging, a nerve biopsy, and genetic tests.
